# Large Language Models Versus Expert Clinicians in Crisis Prediction Among Telemental Health Patients: Comparative Study

**DOI:** 10.2196/58129

**Published:** 2024-08-02

**Authors:** Christine Lee, Matthew Mohebbi, Erin O'Callaghan, Mirène Winsberg

**Affiliations:** 1 Brightside Health San Francisco, CA United States

**Keywords:** mental health, telehealth, PHQ-9, Patient Health Questionnaire-9, suicidal ideation, AI, LLM, OpenAI, GPT-4, generative pretrained transformer 4, tele-mental health, large language model, clinician, clinicians, artificial intelligence, patient information, suicide, suicidal, mental disorder, suicide attempt, psychologist, psychologists, psychiatrist, psychiatrists, psychiatry, clinical setting, self-reported, treatment, medication, digital mental health, machine learning, language model, suicide, crisis, telemental health, tele health, e-health, digital health

## Abstract

**Background:**

Due to recent advances in artificial intelligence, large language models (LLMs) have emerged as a powerful tool for a variety of language-related tasks, including sentiment analysis, and summarization of provider-patient interactions. However, there is limited research on these models in the area of crisis prediction.

**Objective:**

This study aimed to evaluate the performance of LLMs, specifically OpenAI’s generative pretrained transformer 4 (GPT-4), in predicting current and future mental health crisis episodes using patient-provided information at intake among users of a national telemental health platform.

**Methods:**

Deidentified patient-provided data were pulled from specific intake questions of the Brightside telehealth platform, including the chief complaint, for 140 patients who indicated suicidal ideation (SI), and another 120 patients who later indicated SI with a plan during the course of treatment. Similar data were pulled for 200 randomly selected patients, treated during the same time period, who never endorsed SI. In total, 6 senior Brightside clinicians (3 psychologists and 3 psychiatrists) were shown patients’ self-reported chief complaint and self-reported suicide attempt history but were blinded to the future course of treatment and other reported symptoms, including SI. They were asked a simple yes or no question regarding their prediction of endorsement of SI with plan, along with their confidence level about the prediction. GPT-4 was provided with similar information and asked to answer the same questions, enabling us to directly compare the performance of artificial intelligence and clinicians.

**Results:**

Overall, the clinicians’ average precision (0.7) was higher than that of GPT-4 (0.6) in identifying the SI with plan at intake (n=140) versus no SI (n=200) when using the chief complaint alone, while sensitivity was higher for the GPT-4 (0.62) than the clinicians’ average (0.53). The addition of suicide attempt history increased the clinicians’ average sensitivity (0.59) and precision (0.77) while increasing the GPT-4 sensitivity (0.59) but decreasing the GPT-4 precision (0.54). Performance decreased comparatively when predicting future SI with plan (n=120) versus no SI (n=200) with a chief complaint only for the clinicians (average sensitivity=0.4; average precision=0.59) and the GPT-4 (sensitivity=0.46; precision=0.48). The addition of suicide attempt history increased performance comparatively for the clinicians (average sensitivity=0.46; average precision=0.69) and the GPT-4 (sensitivity=0.74; precision=0.48).

**Conclusions:**

GPT-4, with a simple prompt design, produced results on some metrics that approached those of a trained clinician. Additional work must be done before such a model can be piloted in a clinical setting. The model should undergo safety checks for bias, given evidence that LLMs can perpetuate the biases of the underlying data on which they are trained. We believe that LLMs hold promise for augmenting the identification of higher-risk patients at intake and potentially delivering more timely care to patients.

## Introduction

### Background

Suicide is a serious public health concern. Suicide rates have risen at an alarming rate in the past 20 years, and in the United States, suicide is the second leading cause of death in adults aged 18-45 years [1]. In 2021, approximately 50,000 people in the United States died by suicide, which marks the highest national rate of suicide in decades [2]. As suicide rates increase, the behavioral health care workforce in the United States has not expanded enough to keep up with these mental health demands, limiting the timely access to care that is essential for suicide risk detection and prevention [3].

Suicide risk is difficult to predict. Research has demonstrated that there are numerous individual, relationship, community, and societal risk factors associated with suicide, such as history of previous suicide attempts, psychiatric diagnosis, sense of hopelessness, social isolation, community violence, and access to lethal means of suicide [4-9]. More recently, suicide theories and research suggest ideation-to-action pathways to help explain suicide risk, where people who think about suicide are at a higher risk of participating in suicidal behavior [10-13].

The prevalence of suicidal ideation (SI), which is defined as “thinking about, considering, or planning suicide” [14], is common, with 12.3 million Americans aged 18 years and older having thoughts of suicide in 2021 [15]. SI is predictive of suicide attempts and completed suicide [16,17]. SI is also a more sensitive predictor of lifetime risk for suicide than imminent risk [18]. Research has suggested that among those exhibiting SI, there is a 29% conditional probability of making a suicide attempt [19]. Other research has shown that those with nearly daily SI were 5 to 8 times more likely to attempt suicide and 3 to 11 times more likely to die by suicide within 30 days [20].

Artificial intelligence (AI) methods have been used for assessing mental health factors such as psychiatric symptom severity, diagnosis, and clinical risk using free text generated by the patient. Researchers using natural language processing (NLP) and machine learning (ML) were able to identify suicidal behavior from electronic medical records [21] and detect SI in a variety of different free-text settings [22]. In addition, an NLP-based system to determine the likelihood of crisis in patient chat messages to their clinicians was developed and implemented with reliable retrospective and prospective performance as a clinical support tool for a crisis specialist team [23].

Recent advances in AI methods, such as large language models (LLMs), have also shown success in a variety of medical applications. Both generalist LLMs, such as generative pretrained transformer 4 (GPT-4), and medical domain–specific LLMs, such as Med-PaLM 2, have exhibited medical competency on benchmarks such as the United States Medical Licensing Examination (USMLE) exam [24,25]. Generalist LLMs can sometimes outperform the domain-specific LLMs, as was recently found with GPT-4 outperforming MedPaLM 2 on the MedQA medical benchmark [25]. Finally, Med-PaLM-2 was also found to be effective at determining psychiatric functioning from free text, including patient-generated information during patient interviews [26].

### Objective

We seek to leverage the capabilities of LLMs to detect or predict SI with plan among patients enrolled in a national telemental health platform, using patient-generated free text at intake. We will benchmark the performance of this LLM-based prediction against a cohort of senior mental health clinician experts.

## Methods

### Overview

The study consisted of clinicians completing a digital questionnaire where they were asked to predict whether a patient would endorse SI with a plan during the course of their treatment, based on patient-generated text describing their chief complaint. The same chief complaint texts were then served to the LLM GPT-4 with the same questionnaire instructions. The classification performance of the clinicians and GPT-4 were evaluated and compared.

#### Data Acquisition

The retrospective patient data used in this study were collected as part of the standard of care at Brightside Health and deidentified for research purposes. All patients treated at Brightside consent at intake to the terms of use and privacy policy that include consenting to Brightside’s use of their data for research purposes.

#### Inclusion Criteria

Data from patients who completed intake on the Brightside platform after March 15, 2023, and endorsed current SI (at intake) or subsequent SI (post intake and during the course of treatment) were included in the study set, along with a random cohort of patients treated during the same time frame who never endorsed SI with plan. In order to be included in the study sample, patients had to attend at least 1 psychiatric or therapy appointment and complete the chief complaint section of their digital intake form. Patients who left the chief complaint section empty were excluded.

#### Data and Outcome Variables

Patient-generated free text (chief complaint) was extracted from patient intake as the answer to the question “In your own words, what are you feeling or experiencing?” and any personal identifiers (such as age, birthdate, name, location, email address, phone number, and social security number) within the free text were replaced with asterisks. In addition, patient data extracted from intake included age, gender identity, and history of previous suicide attempts. Clinicians and the LLM did not have access to the age or gender identity of the patients and were only shown deidentified patient-generated free text and then the patients’ self-reported history of suicide attempts.

SI with plan was determined from answers to question 9 of the Patient Health Questionnaire-9 (PHQ-9). The PHQ-9 is a self-report questionnaire consisting of 9 questions measuring depression symptom severity ranging from 0 to 3 (not at all, several days, more than half the days, and nearly every day, respectively) within the past 2 weeks and includes a specific question related to the frequency of suicidal thoughts (item 9). If a patient endorses SI on the Brightside platform (item 9 answer value >0), a follow-up Brightside proprietary question asks whether the suicidal thoughts are something the patient has made specific plans for. At Brightside, the PHQ-9 is administered to all patients at intake and requested every 2 weeks during the course of treatment. PHQ-9 answers at intake and the date of the first SI with plan relative to intake were also extracted for this study.

#### Classification Label Definitions

The patients positive for SI with plan were defined as those having endorsed SI in the PHQ-9 at intake or any point during the later course of treatment and subsequently responded that the SI was something they had made specific plans for. Patients negative for SI with plan were defined as those with no PHQ-9 item 9 values >0, that is, those who had never endorsed SI in their PHQ-9 screenings.

#### Clinician Questionnaire Design

After the creation of the study data set, 6 clinicians employed at Brightside Health were recruited and all consented to participate in the study. Clinicians 1, 2, and 3 are senior psychiatrists with 18 to 30 years of clinical experience. Clinicians 4, 5, and 6 are senior psychologists with 10 to 23 years of clinical experience.

Each clinician was presented with the same questionnaire with the same randomized order of questions. Presented with a patient’s deidentified chief complaint, they were asked to answer the following questions: (1) do you expect this patient to endorse SI with a plan in the course of their treatment? (yes or no) and (2) how confident are you? (high, medium, or low).

The clinicians were then presented with additional information as to whether the patient endorsed a previous suicide attempt and reprompted with the same questions. All chief complaints and questions were displayed one at a time. Clinicians were given 2 weeks to complete the questionnaire with no other instructions or training and were not required to complete it in a single sitting.

#### Large Language Model Prompt Design

In this study, we evaluated OpenAI’s GPT-4 text-only model’s ability to predict whether a patient would endorse SI with plan during the course of treatment. All model results were produced in Python (Python Software Foundation) with OpenAI’s application programming interface and GPT-4 (“gpt-4-1106-preview” with default parameters) [27].

First, the model context was constructed using step-by-step instructions and few-shot prompting with 3 real examples of chief complaints and their respective correct, expected answers (Textbox 1). Note that the examples chosen for the few-shot prompting were not included in the study data set and were redacted for publication.

**GPT-4 few-shot prompt template for predicting suicidal ideation with plan in chief complaint text.**

You are a trained psychiatric professional. You will be presented with a patient’s answer to the question “In your own words, what are you feeling or experiencing?” also referred to as a patient’s chief complaint.

Your task is to determine based on a patient’s chief complaint, if that patient will endorse suicidal ideation with plan in the course of their treatment.

You will generate your answer as JSON: {“at_risk”: “”, “confidence”: “”, “risk_indicators”: “[]”}

Use the following step-by-step instructions to determine your answer:

- Step 1. Answer in the key “at_risk” with only the values Yes or No
- Step 2. Answer in the key “confidence” with only the values High, Medium, or Low, how confident you are in your answer.
- Step 3. Answer in the key “risk_indicators” with one or more values from the list of possible risk indicators below that determined your Yes or No answer.

Separate risk indicators with a “; ”.

If Other, specify your answer as “Other-<insert your answer>”. If no risk indicators are identified, answer with None.

List of possible risk indicators values:

- Recent Stress, Loss, or Trauma
- History of Trauma
- Chronic medical conditions
- Substance use
- Previous suicide attempt
- Lack or loss of relationships or support
- Social isolation
- Family history of suicide
- Impulsive or aggressive language
- Explicit mentions of suicide, suicidal thoughts, or self harm
- Death imagery or metaphors
- Apathy, indifference or emotional detachment
- Sense of Hopelessness
- Other

Here is an example of a chief complaint with a Yes to suicidal ideation with plan:

“<*text redacted for publication*> ”

Your answer would be:{“risk_indicators”: “Sense of Hopelessness; Social isolation; Explicit mentions of suicide, suicidal thoughts, or self harm”, “at_risk”: “Yes”, “confidence”: “High”}

Here is an example of a chief complaint with a No to suicidal ideation with plan: “<*text redacted for publication*>”

Your answer would be: {“risk_indicators”: “None”, “at_risk”: “No”, “confidence”: “High”}

Here is an example of a chief complaint with a No to suicidal ideation with plan:

“<*text redacted for publication*>”

Your answer would be: {“risk_indicators”: “None”, “at_risk”: “No”,“confidence”: “High”}

Next, the output format of the model was specified as JavaScript Object Notation for ease of analysis. In addition to the prediction of SI with plan during the course of treatment, the model was also asked to provide a confidence level (high, medium, and low) to the prediction (similar to the clinicians’ questionnaire) and to provide reasoning from a list of explicitly provided risk indicators.

Finally, the deidentified patient-generated chief complaint text was given to the model in the user prompt. Each chief complaint was provided independently and then the LLM was reset back to the original context.

In order to evaluate the model’s performance when served the additional information of patient self-reported previous suicide attempts, the sentence “I have attempted suicide before” or “I have never attempted suicide before” was appended to the end of the chief complaint and served as the prompt with the same context.

#### Performance Analysis

All analyses were performed in Python 3.8.12 with the package scikit-learn version 1.3.1 [28]. For comparison of performance, analyses were performed on positive for SI with plan at intake versus negative for SI during the entire course of treatment, as well as positive for SI with plan post intake versus the same data set of negative for SI during treatment.

#### Classification and Predictive Performance

Clinician and model performances in the ability to predict whether a chief complaint text sample was positive for SI with plan, at intake, and post intake, were evaluated for accuracy, sensitivity, specificity, and precision. Accuracy was defined as the proportion of correctly predicted samples over the total number of samples. Precision (or positive predictive value) was defined as the proportion of correctly predicted positive samples over the total number of predicted positive samples. Sensitivity was defined as the proportion of correctly predicted positive samples over the total number of positive samples. Specificity was defined as the proportion of correctly predicted negative samples over the total number of negative samples. As an additional baseline reference, previous suicide attempt information (yes or no) as a stand-alone predictor was also included in the evaluation.

#### Clinician and Large Language Model Agreement

To measure the agreement between the clinician and GPT-4’s predictions, the Cohen κ statistic, which measures interrater agreement for categorical data, was calculated for each clinician and GPT-4 pairing.

#### Clinical Consensus and Confidence

Clinical consensus was defined as instances in which all clinicians answered with the same predicted outcome for a given sample, regardless of whether the prediction was correct. Rates of clinical consensus and rates of confidence were calculated to measure the variability and difficulty of clinical assessments on the given samples.

### Accuracy of Clinical Consensus Influence on Large Language Model Performance

To measure the influence of the accuracy of clinical consensus on GPT-4 performance, subsets of chief complaint text samples where at least 1, 2, 3, 4, 5, or all 6 clinicians not only agreed but also correctly predicted the outcome for a given sample were evaluated for GPT-4 accuracy, sensitivity, specificity, and precision.

### Risk Indicator Language and Clinician Performance

The GPT-4 prompt included a request to provide the rationale for its prediction from a list of explicitly provided risk indicators (Textbox 1). Clinician performance was then re-evaluated on patient chief complaints with no GPT-4–identified risk indicators as a way to understand how difficult these cases were to clinical experts.

Due to the generative nature of an LLM, GPT-4 occasionally will produce an answer that is not from the list of those that are explicitly defined in the instructions. For the purpose of this analysis, only the following explicit risk indicators defined as exact string match were assessed: “recent stress, loss, or trauma,” “history of trauma,” “chronic medical conditions,” “substance use,” “previous suicide attempt,” “lack or loss of relationships or support,” “social isolation,” “family history of suicide,” “impulsive or aggressive language,” “explicit mentions of suicide, suicidal thoughts, or self-harm,” “death imagery or metaphors,” “apathy, indifference or emotional detachment,” and “sense of hopelessness.”

### Ethical Considerations

This study was conducted according to the guidelines of the Declaration of Helsinki and approved by the Institutional Review Board of WCG (protocol 20240207).

## Results

### Overview

At the conclusion of the study (December 13, 2023), 260 patients met inclusion criteria and were positive for SI with plan. A total of 140 patients were positive for SI with plan at the time of intake and 120 patients were positive for SI with plan post intake in their subsequent treatment. A random subset of 200 patients was selected from those who met the inclusion criteria and were negative for SI with plan. A summary of the data can be found in Table 1.

**Table 1:** Summary of data for patients with no SI with plan (n=200), SI with plan indicated at intake (n=140), and SI with plan indicated post intake (n=120).

		No SI with plan (n=200)	SI with plan at intake (n=140)	SI with plan post intake (n=120)
Age (years), mean (95% CI)		37.2 (35.7-38.9)	34.4 (32.5-36.3)	32.4 (30.3-34.5)
**Gender identity, n (%)**				
	Women	135 (67.5)	76 (54.3)	59 (49.2)
	Men	64 (32)	57 (40.7)	59 (49.2)
**Ethnicity, n (%)**				
	White	152 (76)	94 (67.1)	73 (60.8)
	Hispanic	16 (8)	20 (14.3)	14 (11.7)
	Black	13 (6.5)	13 (9.3)	16 (13.3)
	Asian	10 (5)	6 (4.3)	8 (6.7)
	Other	9 (4.5)	7 (5)	9 (7.5)
Average chief complaint word count (95% CI)		49.6 (41.3-57.9)	58 (33-83.1)	57.2 (44.2-70.3)
Average days between first SI with plan date and chief complaint (95% CI)		—^a^	0 (0)	62.6 (52.4-72.8)
Average PHQ-9^b^ total score at first SI with plan (95% CI)		—	21.1 (20.2-21.9)	19.0 (17.8-20.2)
**Number of patients with PHQ-9 item 9 score value at first SI with plan, n (%)**				
	0	—	0 (0)	0 (0)
	1	—	32 (22.9)	34 (28.3)
	2	—	34 (24.3)	29 (24.2)
	3	—	74 (52.9)	57 (47.5)
	With specific plan	—	140 (100)	120 (100)
Average PHQ-9 total score at intake (95% CI)		13.5 (12.7-14.2)	20.9 (20.1-21.7)	18.3 (17.2-19.4)
**Number of patients with PHQ-9 item 9 score value at intake, n (%)**				
	0	200 (100)	0 (0)	34 (28.3)
	1	0 (0)	32 (22.9)	34 (28.3)
	2	0 (0)	34 (24.3)	20 (16.7)
	3	0 (0)	74 (52.9)	32 (26.7)
	With specific plan	0 (0)	140 (100)	0 (0)
	Previous suicide attempt	14 (7)	55 (39.3)	40 (33.3)

^a^Not applicable.

^b^PHQ: Patient Health Questionnaire-9.

### Prediction Performance

#### Predicting SI With Plan at Intake

The performance of the previous suicide attempt alone to predict SI with plan at the time of intake was similar to both GPT-4 and clinicians except for the low sensitivity at 0.39 (Table 2).

GPT-4 performed with similar accuracy (0.67) and higher sensitivity (0.62) in predicting SI with plan at the time of intake based on the chief complaint text only, as compared with the average accuracy (0.7) and sensitivity (0.53) across our 6 clinician participants (Table 2). However, GPT-4 performed with lower specificity (0.71) and precision (0.6) than the average clinician specificity (0.82) and precision (0.69). The interrater agreement between GPT-4 and each clinician was moderate as indicated by an average Cohen κ of 0.49.

Additional knowledge of the previous suicide attempt increased overall performance across clinicians (accuracy=0.75; sensitivity=0.59; specificity=0.86; precision=0.77). Additional knowledge of the previous suicide attempts significantly increased sensitivity for GPT-4 but decreased accuracy, specificity, and precision (accuracy=0.64; sensitivity=0.84; specificity=0.51; precision=0.54). The interrater agreement between GPT-4 and each clinician also decreased to an average Cohen κ of 0.39 with the additional information of the previous suicide attempts.

**Table 2:** Performance results for predicting suicidal ideation with a plan at the time of intake and predicting suicidal ideation with a plan in the future post intake based solely on chief complaint versus chief complaint plus knowledge of the previous attempt for GPT-4 and 6 clinicians. The performance of the previous suicide attempt alone as a predictor is included for baseline reference.

				True negative, n		False positive, n		False negative, n		True positive, n		Accuracy		Sensitivity		Specificity		Precision		Cohen κ with GPT-4
**SI with plan at intake (n=140) versus no SI with plan (n=200)**																				
	Baseline for comparison: previous suicide attempts only		186		14		85		55		0.71		0.39		0.93		0.8		—^a^	
	**Chief complaint text only**																			
		GPT-4	141		59		53		87		0.67		0.62		0.71		0.6		—	
		Clinician 1	160		40		58		82		0.71		0.59		0.8		0.67		0.53	
		Clinician 2	189		11		95		45		0.69		0.32		0.95		0.80		0.36	
		Clinician 3	138		62		48		92		0.68		0.66		0.69		0.6		0.56	
		Clinician 4	183		17		85		55		0.77		0.39		0.92		0.76		0.44	
		Clinician 5	162		38		58		82		0.72		0.59		0.81		0.68		0.5	
		Clinician 6	156		44		52		88		0.72		0.63		0.78		0.67		0.54	
		Average across clinicians	—		—		—		—		0.70		0.53		0.82		0.7		0.49	
	**Chief complaint text + previous suicide attempt knowledge**																			
		GPT-4	102		98		23		117		0.64		0.84		0.51		0.54		—	
		Clinician 1	163		37		49		91		0.75		0.65		0.82		0.71		0.46	
		Clinician 2	194		6		89		51		0.72		0.36		0.97		0.9		0.21	
		Clinician 3	152		48		39		101		0.74		0.72		0.76		0.68		0.5	
		Clinician 4	187		13		67		73		0.77		0.52		0.94		0.85		0.329	
		Clinician 5	173		27		53		87		0.77		0.62		0.87		0.76		0.4	
		Clinician 6	159		41		47		93		0.74		0.66		0.8		0.69		0.42	
		Average across clinicians	—		—		—		—		0.75		0.59		0.86		0.77		0.39	
**SI with plan post intake (n=120) versus no SI with plan (n=200)**																				
	Baseline for comparison: prior suicide attempt only		186		14		80		40		0.71		0.33		0.93		0.74		—	
	**Chief complaint text only**																			
		GPT-4	141		—		65		55		0.61		0.46		0.71		0.48		—	
		Clinician 1	160		—		69		51		0.66		0.43		0.8		0.56		0.44	
		Clinician 2	189		—		100		20		0.65		0.17		0.95		0.65		0.26	
		Clinician 3	138		—		54		66		0.64		0.55		0.69		0.52		0.44	
		Clinician 4	183		—		84		36		0.68		0.3		0.92		0.68		0.34	
		Clinician 5	162		—		70		50		0.66		0.42		0.81		0.57		0.43	
		Clinician 6	156		—		56		64		0.69		0.53		0.78		0.59		0.50	
		Average across clinicians	—		—		—		—		0.66		0.4		0.82		0.59		0.4	
	**Chief complaint text + prior suicide attempt knowledge**																			
		GPT-4	102		—		31		89		0.6		0.74		0.51		0.48		—	
		Clinician 1	163		—		59		61		0.7		0.51		0.82		0.62		0.37	
		Clinician 2	194		—		90		30		0.7		0.25		0.97		0.83		0.17	
		Clinician 3	152		—		49		71		0.7		0.59		0.76		0.6		0.45	
		Clinician 4	187		—		76		44		0.72		0.37		0.94		0.77		0.27	
		Clinician 5	173		—		63		57		0.72		0.48		0.87		0.68		0.36	
		Clinician 6	159		—		54		66		0.7		0.55		0.8		0.62		0.35	
		Average across clinicians	—		—		—		—		0.71		0.46		0.86		0.69		0.33	

^a^Not applicable.

#### Predicting SI With Plan Post Intake

Performance decreased for both clinicians and GPT-4 when predicting future SI with plan post intake. Note that specificity results were consistent with predicting SI with plan at intake, as there was no change in the negative samples.

GPT-4 performed with similar accuracy (0.61) and higher, but still poor, sensitivity (0.46) in predicting SI with plan post intake based solely on the chief complaint compared with the average accuracy (0.66) and sensitivity (0.4) across the 6 clinicians (Table 2). GPT-4 performed with lower precision (0.48) than the average clinician precision (0.59). The interrater agreement between GPT-4 and each clinician remained moderate at an average Cohen κ of 0.4.

Additional knowledge of the previous suicide attempts increased performance across all clinicians (accuracy=0.71; sensitivity=0.46; precision=0.69). Additional knowledge of the previous suicide attempt significantly increased sensitivity for GPT-4 but decreased accuracy and precision (accuracy=0.6; sensitivity=0.74; precision=0.48). The interrater agreement between GPT-4 and each clinician was lower, with an average Cohen κ of 0.33 with the additional information.

### Clinical Consensus and Confidence

Clinical consensus was defined as instances in which all 6 clinicians agreed on the predicted outcome for a given sample, regardless of whether the prediction was correct. Clinical consensus occurred in 52% (104/200) of “no SI with plan” samples, 40.7% (57/140) of “SI with plan at intake” samples, and 40% (48/120) of “SI with plan postintake” samples (Table 3). For SI with plan samples with a clinical consensus, the agreed-upon prediction was correct 61.4% (35/140) of the time for “SI with plan at intake” versus much lower at 25% (25/120) of the time for “SI with plan postintake.” For the “no SI with plan” samples, the clinicians’ agreed-upon prediction was correct at a high rate of 98.1% (102/200).

**Table 3:** Rates of clinical consensus are defined as instances in which all 6 clinicians agreed on the predicted outcome for a given sample.

	No SI with plan (n=200), n (%)	SI with plan at intake (n=140), n (%)	SI with plan post intake (n=120), n (%)
Number of samples with clinical consensus	104 (52)	57 (40.7)	48 (40)
Clinical consensus predicted SI with plan	2 (1.9)	35 (61.4)	12 (25)
Clinical consensus predicted no SI with plan	102 (98.1)	22 (38.6)	36 (75)

In addition, clinicians, on average, had lower rates of high confidence (even when answers were correct) compared with GPT-4 (Table 4). On average, clinicians answered correctly “no with high confidence” in 9.5% (19/200) of “no SI with plan” samples versus GPT-4 answered “no with high confidence” in 35% (70/200). Clinicians answered correctly “yes with high confidence” in 15.7% (22/140) of “SI with plan at intake” samples versus GPT-4 at 29.3% (41/140). Rates of correctly answered “yes with high confidence” were lower in “SI with plan postintake” samples but were higher for GPT-4 compared with average clinician rates (13.3%, 16/120 vs 7.2%, 8.7/120).

**Table 4:** Rates of high confidence answers.

	No SI^a^ with plan (n=200)			SI with plan at intake (n=140)			SI with plan post intake (n=120)	
	Answered yes with high confidence, n (%)	Answered no with high confidence, n (%)	Answered yes with high confidence, n (%)		Answered no with high confidence, n (%)	Answered yes with high confidence, n (%)		Answered no with high confidence, n (%)
Clinician 1	5 (2.5)	6 (3)	45 (32.1)		1 (0.7)	16 (13.3)		2 (1.7)
Clinician 2	0 (0)	19 (9.5)	5 (3.6)		7 (5.0)	1 (0.8)		4 (3.3)
Clinician 3	2 (1)	41 (20.5)	20 (14.3)		9 (6.4)	9 (7.5)		6 (5)
Clinician 4	0 (0)	0 (0)	1 (0.7)		0 (0)	0 (0)		0 (0)
Clinician 5	0 (0)	2 (1)	23 (16.4)		0 (0)	5 (4.2)		3 (2.5)
Clinician 6	2 (1)	46 (23)	38 (27.1)		13 (9.3)	21 (17.5)		12 (10)
Average across clinicians (%)	1.5 (0.75)	19 (9.5)	22 (15.7)		5 (3.6)	8.7 (7.2)		4.5 (3.8)
GPT-4^b^	1 (0.5)	70 (35.0)	41 (29.3)		17 (12.1)	16 (13.3)		14 (11.7)

^a^SI: suicidal ideation.

^b^GPT-4: generative pretrained transformer 4.

### Accuracy of Clinical Consensus and GPT-4 Performance

A range of accurate clinical consensus samples was defined as samples where several clinicians, ranging from at least 1 to all 6, not only agreed on the predicted outcome but also correctly predicted the outcome. There were 316 samples of the “SI with plan at intake” and “no SI with plan” samples where at least 1 clinician predicted the outcome correctly versus 137 samples where all 6 clinicians predicted the outcome correctly (Table 5). There were 282 samples of the “SI with plan postintake” and “no SI with plan” samples where at least 1 clinician predicted the outcome correctly versus 114 samples where all 6 clinicians predicted the outcome correctly.

**Table 5:** Performance results for GPT-4 solely on the chief complaint in samples where at least 1, 2, 3, 4, 5, or all 6 clinicians correctly predicted the outcome of those samples.

Number of clinicians correctly predicting samples’ consensus threshold			Number of samples		True negative			False positive			False negative			True positive			Accuracy			Sensitivity			Specificity			Precision
**SI with plan at intake (original n=140) versus no SI with plan (original n=200)**																										
	≥1	316		141			57			32			86			0.72			0.73			0.71			0.60	
	≥2	284		141			52			14			77			0.77			0.85			0.73			0.60	
	≥3	259		137			42			7			73			0.81			0.91			0.77			0.64	
	≥4	236		133			36			2			65			0.84			0.97			0.79			0.64	
	≥5	200		123			24			0			53			0.88			1			0.84			0.69	
	6	137		89			13			0			35			0.91			1			0.87			0.73	
**SI with plan post intake (original n=120) versus no SI with plan (original n=200)**																										
	≥1	282		141		57			31			53			0.69			0.63			0.71			0.48		
	≥2	266		141		52			23			50			0.72			0.69			0.73			0.49		
	≥3	233		137		42			10			44			0.78			0.82			0.77			0.51		
	≥4	211		133		36			6			36			0.80			0.86			0.79			0.5		
	≥5	169		123		24			1			21			0.85			0.96			0.84			0.47		
	6	114		89		13			0			12			0.89			1			0.87			0.48		

As the accurate clinical consensus threshold increased, GPT-4 performance increased significantly in those samples (Table 5). When assessing the “SI with plan at intake” and “no SI with plan” samples with a clinical consensus of 3 or more and correct predictions, GPT-4 performed with an accuracy of 0.81, sensitivity of 0.91, specificity of 0.77, and precision of 0.64. When assessing the “SI with plan postintake” and “no SI with plan” samples with a clinical consensus of 3 or more and correct predictions, GPT-4 performed with an accuracy of 0.80, sensitivity of 0.86, and precision of 0.51.

### Risk Indicators Identified in Chief Complaint Text by GPT-4

At least 1 risk indicator was identified in the chief complaint text by GPT-4 on 45.5% (91/200) of “no SI with plan” samples (Table 6). A total of 70% (98/140) of “SI with plan at intake” samples and 54.2% (65/120) of “SI with plan postintake” samples had at least 1 GPT-4–identified risk indicator. The most common risk indicator in “SI with plan at intake” samples identified by GPT-4 was “sense of hopelessness” (in 40% [56/140] of samples, compared with 27.5% [33/120] of “SI with plan postintake” and 16.5% [33/200] of “no SI with plan”). The most common risk indicator in “no SI with plan” samples was “recent stress, loss, or trauma” (in 25.5% [51/200] of samples, compared with 22.1% [31/140] of “SI with plan at intake” samples and 17.5% [21/120] of “SI with plan postintake” samples). In addition, the rate of identification of “social isolation” as a risk factor in “SI with plan postintake” samples (15/120, 12.5%) was higher in both “no SI with plan” (22/140, 5.7%) samples and “SI with plan at intake” samples (33/200, 6.5%).

**Table 6:** Number of samples per explicit risk indicator identified by GPT-4.

		No SI with plan (n=200)	SI with plan at intake (n=140)	SI with plan post intake (n=120)
**Number of risk indicators identified by GPT-4, n (%)**				
	0	109 (54.5)	42 (30)	55 (45.8)
	1	34 (17)	28 (20	22 (18.3)
	2	34 (17)	37 (26.4)	18 (15)
	3	16 (8)	22 (15.7)	15 (12.5)
	4	4 (2)	6 (4.3)	8 (6.7)
	5	3 (1)	3 (2.1)	1 (0.8)
	6	0 (0)	2 (1.4)	1 (0.8)
**Risk indicator identified by GPT-4, n (%)**				
	Sense of hopelessness	33 (16.5)	56 (40)	33 (27.5)
	Explicit mentions of suicide, suicidal thoughts, or self-harm	2 (1)	38 (27.1)	19 (15.8)
	Recent stress, loss, or trauma	51 (25.5)	31 (22.1)	21 (17.5)
	Apathy, indifference, or emotional detachment	19 (9.5)	22 (15.7)	19 (15.8)
	Lack or loss of relationships or support	22 (11)	17 (12.1)	12 (10)
	Social isolation	13 (6.5)	8 (5.7)	15 (12.5)
	Chronic medical conditions	13 (6.5)	13 (9.3)	8 (6.7)
	History of trauma	13 (6.5)	10 (7.1)	8 (6.7)
	Impulsive or aggressive language	3 (1.5)	8 (5.7)	6 (5)
	Previous suicide attempt	0 (0)	9 (6.4)	1 (0.8)
	Substance use	10 (5)	6 (4.3)	3 (2.5)
	Family history of suicide	0 (0)	0 (0)	1 (0.8)
	Death imagery or metaphors	2 (1)	1 (0.7)	0 (0)

### Chief Complaints With No Risk Indicators and Clinician Performance

Assessing the clinicians’ performance on samples where GPT-4 identified no explicit risk indicators in the chief complaint text, the average clinician sensitivity was found to be low for both “SI with plan at intake” and “SI with plan postintake” at 0.22 and 0.17, respectively (Table 7). The average clinician specificity and precision were high for both “SI with plan at intake” and “SI with plan postintake” at 0.93 and 0.63 versus 0.93 and 0.6, respectively. While the sample size in this analysis was significantly decreased, n=109/200 (54.5%) for “no SI with plan,” n=42/140 (30%) for “SI with plan at intake,” and n=55/120 (45.8%) for “SI with plan postintake,” clinicians’ performance resulted in fewer false positives and a lower rate of positive prediction, indicating that clinicians are less likely to predict SI with plan in patients where GPT did not identify any risk factors.

**Table 7:** Performance results for chief complaint text-only samples where GPT-4 identified zero explicit risk indicators.

		True negative	False positive	False negative	True positive	Accuracy	Sensitivity	Specificity	Precision
**SI^a^ with plan at intake (n=42) versus no SI with plan (n=109)**									
	GPT-4	109	0	40	2	0.74	0.05	1	1
	Clinician 1	100	9	33	9	0.72	0.21	0.92	0.5
	Clinician 2	108	1	40	2	0.73	0.05	0.99	0.67
	Clinician 3	91	18	27	15	0.70	0.36	0.84	0.46
	Clinician 4	109	0	39	3	0.74	0.07	1	1
	Clinician 5	101	8	28	14	0.76	0.33	0.93	0.64
	Clinician 6	96	13	29	13	0.72	0.31	0.88	0.5
	Average across clinicians	—^b^	—	—	—	0.73	0.22	0.93	0.63
**SI with plan post intake (n=55) versus no SI with plan (n=109)**									
	GPT-4	109	0	54	1	0.67	0.02	1	1
	Clinician 1	100	9	45	10	0.67	0.18	0.92	0.53
	Clinician 2	108	1	54	1	0.67	0.02	0.99	0.5
	Clinician 3	91	18	37	18	0.67	0.33	0.84	0.5
	Clinician 4	109	0	51	4	0.69	0.07	1	1
	Clinician 5	101	8	44	11	0.68	0.2	0.93	0.58
	Clinician 6	96	13	43	12	0.66	0.22	0.88	0.48
	Average across clinicians	—	—	—	—	0.67	0.17	0.93	0.6

^a^SI: suicidal ideation.

^b^Not applicable.

## Discussion

### Overview

The objective of this study was to evaluate the performance of the foundation LLM GPT-4 compared with experienced mental health clinicians in predicting SI with plan based on a patient-generated chief complaint–free text at intake on a national telemental health platform. This study supports previous research that LLMs are able to perform comparably to clinicians in medical applications and that generalist models such as GPT-4 are able to deliver comparable performance without specialized fine-tuning or domain expertise [24,25].

### Findings

GPT-4 is capable of predicting the risk of SI with plan using patient-generated chief complaint–free text without extensive work on prompt design and without being trained explicitly on this task. The performance of these GPT-4–based predictions approach those of the clinicians on a variety of measures.

The variability in clinicians’ performance and agreement indicate that identifying SI with plan in patient text alone is a difficult problem even for clinical experts. However, using the clinical experts in this study as a benchmark, GPT-4 was still able to perform comparably in sensitivity but with lower specificity and precision. When assessing GPT-4 on samples with high clinician agreement and performance, this study found that GPT-4 was capable of significantly high sensitivity as well as specificity. These results support that models such as GPT-4, without large amounts of time spent on highly complex data cleaning or model training, are capable of identifying the risk of crisis comparable to the average clinician.

This study also explored the use of GPT-4 as an NLP technique for the extraction of meaningful clinical information. GPT-4 was able to identify and return explicit indicators of risk in text, such as “sense of hopelessness,” that could further assist in crisis triaging and resourcing.

In addition, while not a specific aim or analysis in this study, the average clinician took approximately 3 hours to evaluate the 460 samples of text provided. GPT-4 completed the full evaluation in less than 10 minutes, without optimization for computing or memory, highlighting the possible increased operational efficiency that could be leveraged by automating a tedious and emotionally trying manual task.

Taking into consideration the current behavioral health care workforce shortage, and the increasing rates of suicide, there is a need for scalable, efficient, technology-enabled screening techniques, such as the one used in this study, to assist with suicide risk detection. More efficient risk detection will allow for faster delivery of interventions to help prevent suicide attempts. The use of technology for this purpose would also be a cost-saving and efficient way to more broadly screen for suicide risk. Patients deemed at high risk might be triaged by clinicians with greater expertise in managing suicidality.

Responsible integration and the use of generative AI as a screening tool for predicting the likelihood of crisis would depend on achieving at least similar accuracy to a team of clinicians and should always follow-up with a clinician review, who would be given additional context behind the GPT-4–based prediction and have access to additional clinical data.

Overall, GPT-4 shows promise as a solution to help clinicians deliver more timely care.

### Limitations

We do not intend for this study, the LLM choice, or the prompt design to be viewed as a generalizable solution to predict and identify suicidal risk. Instead, we have shown how the capabilities of these LLMs can be tailored to specific psychiatric assessments and how they compare to the limitations of expert clinician predictions. We hope that the findings encourage further research.

Several limitations in this study must be addressed before the results of such a system could be applied in practice, including but not limited to data from a larger or more diverse population, use of other LLMs, and in particular, LLMs that were built for application in the medical domain, and a greater exploration of prompt design and its impact on performance. Similar to the use of real-time clinical decision support for precision prescribing at Brightside, which is reliant on medical decision-making by trained clinicians, the use of LLM for triage would be limited to suggestions and distillation of information for further clinician assessment [29].

Suicide has been notoriously difficult to predict. Due to the difficult nature of identifying or predicting future SI with plan, precision uncertainties are a reality in treating higher-severity behavioral health patients. This can be seen by the number of false positives and lower precision across several clinicians. Due to this uncertainty, awareness of risk does not necessarily dictate treatment decisions but might influence triage to a provider with more expertise in treating suicidality.

GPT-4 was on the higher end for false positives with chief complaint text only relative to the clinicians, and when previous attempt knowledge was added, this rate was almost doubled, making this metric relative to the worst-performing clinician. While work should be done to further align this GPT-4–based system with the expert clinicians, especially with previous attempt information, these false positives are clearly a reality in treating patients today.

GPT-4 was on the lower end for false negatives relative to the clinicians, in some cases having half as many false positives as the worst-performing clinicians. It is our view that increasing awareness around potential risk through the use of systems such as this is valuable, especially for clinicians who have less expertise.

Finally, as previously discussed, LLMs have tendencies to perpetuate biases inherent in the data on which they are trained [30]. Future work should explore how these biases may influence the quality of the prediction within different subpopulations of patients [31].

### Conclusions

The use of ML and LLMs to analyze speech and language patterns offers an opportunity for behavioral health clinicians and researchers to explore technologies such as these to assist with the detection and prediction of mental health conditions, along with specific symptoms such as suicidal thoughts, intent, and behaviors [32]. This study served as a model for comparing the predictive value of generative AI to clinician (imperfect) predictions when both were given access to the same limited data set. Research evaluating applications of AI technology to human speech, language, and behavior is in its infancy, but findings such as the ones presented in this study may help clinicians and researchers leverage the potential of LLMs to help those struggling with mental illness. Generative AI has the potential to transform areas of mental health care that might otherwise be overlooked. However, great care must be taken by both developers of this technology and the clinicians who deploy them to ensure that the benefits far outweigh the safety challenges and risks.

Further research is encouraged in this area, with consideration of the ethical and clinical implications of the use of AI for detecting and predicting mental health issues [32]. This research will assist in setting standards and guidelines for how such use could be deployed.

## Abbreviations

AI: artificial intelligence
GPT-4: generative pretrained transformer 4
LLM: large language model
ML: machine learning
NLP: natural language processing
PHQ-9: Patient Health Questionnaire-9
SI: suicidal ideation
USMLE: United States Medical Licensing Examination
